# Supplementary material for: Role and mechanism of NCAPD3 in promoting malignant behaviors in gastric cancer
Source: Front Pharmacol. 2024 Apr 22;15:1341039. doi: 10.3389/fphar.2024.1341039 (PMC11070777; doi:10.3389/fphar.2024.1341039)
Supplement: Supplementary file 11 [file DataSheet2.ZIP › GSEA/Oncogenic signature/my_analysis.Gsea.1599461962066/STK33_NOMO_UP.html]

Details for gene set STK33\_NOMO\_UP[GSEA]

|  || Dataset | filtered\_dataset.sample\_info.cls#WT\_versus\_NCAPD3\_MUT |
| Phenotype | sample\_info.cls#WT\_versus\_NCAPD3\_MUT |
| Upregulated in class | NCAPD3\_MUT |
| GeneSet | STK33\_NOMO\_UP |
| Enrichment Score (ES) | -0.33102742 |
| Normalized Enrichment Score (NES) | -2.044571 |
| Nominal p-value | 0.0 |
| FDR q-value | 0.022286851 |
| FWER p-Value | 0.1 |
Table: GSEA Results Summary

  

Fig 1: Enrichment plot: STK33\_NOMO\_UP      
 Profile of the Running ES Score & Positions of GeneSet Members on the Rank Ordered List

  

| SYMBOL | TITLE | RANK IN GENE LIST | RANK METRIC SCORE | RUNNING ES | CORE ENRICHMENT || 1 | 23174 | ZCCHC14 | 27 | 1.004 | 0.0142 | No |
| 2 | 23253 | ANKRD12 | 34 | 0.977 | 0.0429 | No |
| 3 | 55727 | BTBD7 | 38 | 0.954 | 0.0730 | No |
| 4 | 2921 | CXCL3 | 57 | 0.884 | 0.0898 | No |
| 5 | 3667 | IRS1 | 83 | 0.821 | 0.0993 | No |
| 6 | 817 | CAMK2D | 165 | 0.710 | 0.0640 | No |
| 7 | 9778 | KIAA0232 | 247 | 0.633 | 0.0260 | No |
| 8 | 161582 | DYX1C1 | 253 | 0.626 | 0.0436 | No |
| 9 | 22845 | DOLK | 269 | 0.618 | 0.0535 | No |
| 10 | 1601 | DAB2 | 327 | 0.577 | 0.0313 | No |
| 11 | 2919 | CXCL1 | 405 | 0.534 | -0.0071 | No |
| 12 | 2920 | CXCL2 | 454 | 0.501 | -0.0253 | No |
| 13 | 9690 | UBE3C | 470 | 0.493 | -0.0196 | No |
| 14 | 84159 | ARID5B | 699 | 0.379 | -0.1739 | No |
| 15 | 6385 | SDC4 | 871 | -0.282 | -0.2897 | No |
| 16 | 220213 | OTUD1 | 889 | -0.304 | -0.2919 | No |
| 17 | 54532 | USP53 | 898 | -0.322 | -0.2868 | No |
| 18 | 4973 | OLR1 | 944 | -0.366 | -0.3074 | No |
| 19 | 10123 | ARL4C | 957 | -0.377 | -0.3035 | No |
| 20 | 3176 | HNMT | 991 | -0.398 | -0.3142 | No |
| 21 | 3914 | LAMB3 | 1015 | -0.414 | -0.3170 | Yes |
| 22 | 2673 | GFPT1 | 1016 | -0.414 | -0.3030 | Yes |
| 23 | 57552 | NCEH1 | 1020 | -0.419 | -0.2910 | Yes |
| 24 | 23764 | MAFF | 1039 | -0.436 | -0.2894 | Yes |
| 25 | 80315 | CPEB4 | 1053 | -0.448 | -0.2838 | Yes |
| 26 | 65981 | CAPRIN2 | 1064 | -0.455 | -0.2757 | Yes |
| 27 | 6016 | RIT1 | 1072 | -0.458 | -0.2653 | Yes |
| 28 | 7378 | UPP1 | 1111 | -0.484 | -0.2768 | Yes |
| 29 | 2012 | EMP1 | 1119 | -0.494 | -0.2652 | Yes |
| 30 | 604 | BCL6 | 1136 | -0.504 | -0.2598 | Yes |
| 31 | 153222 | CREBRF | 1187 | -0.559 | -0.2775 | Yes |
| 32 | 9975 | NR1D2 | 1230 | -0.605 | -0.2878 | Yes |
| 33 | 2005 | ELK4 | 1235 | -0.612 | -0.2700 | Yes |
| 34 | 114907 | FBXO32 | 1248 | -0.625 | -0.2576 | Yes |
| 35 | 22822 | PHLDA1 | 1255 | -0.643 | -0.2403 | Yes |
| 36 | 135114 | HINT3 | 1257 | -0.645 | -0.2191 | Yes |
| 37 | 11057 | ABHD2 | 1258 | -0.646 | -0.1972 | Yes |
| 38 | 9262 | STK17B | 1268 | -0.659 | -0.1815 | Yes |
| 39 | 57561 | ARRDC3 | 1324 | -0.744 | -0.1966 | Yes |
| 40 | 54742 | LY6K | 1331 | -0.755 | -0.1754 | Yes |
| 41 | 5997 | RGS2 | 1336 | -0.770 | -0.1523 | Yes |
| 42 | 57568 | SIPA1L2 | 1347 | -0.788 | -0.1329 | Yes |
| 43 | 9966 | TNFSF15 | 1376 | -0.900 | -0.1229 | Yes |
| 44 | 9990 | SLC12A6 | 1379 | -0.922 | -0.0932 | Yes |
| 45 | 8867 | SYNJ1 | 1387 | -0.979 | -0.0651 | Yes |
| 46 | 26959 | HBP1 | 1395 | -1.073 | -0.0339 | Yes |
| 47 | 4312 | MMP1 | 1407 | -1.304 | 0.0022 | Yes |
Table: GSEA details [plain text format]

  

Fig 2: STK33\_NOMO\_UP      
 Blue-Pink O' Gram in the Space of the Analyzed GeneSet

  

Fig 3: STK33\_NOMO\_UP: Random ES distribution      
 Gene set null distribution of ES for **STK33\_NOMO\_UP**
